# Comparison of Pheochromocytoma-Specific Morbidity and Mortality Among Adults With Bilateral Pheochromocytomas Undergoing Total Adrenalectomy vs Cortical-Sparing Adrenalectomy

**DOI:** 10.1001/jamanetworkopen.2019.8898

**Published:** 2019-08-09

**Authors:** Hartmut P. H. Neumann, Uliana Tsoy, Irina Bancos, Vincent Amodru, Martin K. Walz, Amit Tirosh, Ravinder Jeet Kaur, Travis McKenzie, Xiaoping Qi, Tushar Bandgar, Roman Petrov, Marina Y. Yukina, Anna Roslyakova, Anouk N. A. van der Horst-Schrivers, Annika M. A. Berends, Ana O. Hoff, Luciana Audi Castroneves, Alfonso Massimiliano Ferrara, Silvia Rizzati, Caterina Mian, Sarka Dvorakova, Kornelia Hasse-Lazar, Andrey Kvachenyuk, Mariola Peczkowska, Paola Loli, Feyza Erenler, Tobias Krauss, Madson Q. Almeida, Longfei Liu, Feizhou Zhu, Mònica Recasens, Nelson Wohllk, Eleonora P. M. Corssmit, Zulfiya Shafigullina, Jan Calissendorff, Simona Grozinsky-Glasberg, Tada Kunavisarut, Camilla Schalin-Jäntti, Frederic Castinetti, Petr Vlček, Dmitry Beltsevich, Viacheslav I. Egorov, Francesca Schiavi, Thera P. Links, Ronald M. Lechan, Birke Bausch, William F. Young, Charis Eng

**Affiliations:** 1 Section of Preventive Medicine, Medical Center–University of Freiburg, Faculty of Medicine, Albert-Ludwig-University Freiburg, Freiburg, Germany; 2 Neuroendocrinology Laboratory, Endocrinology Institute, Almazov National Medical Research Centre, St Petersburg, Russia; 3 Division of Endocrinology, Diabetes, Metabolism, and Nutrition, Mayo Clinic, Rochester, Minnesota; 4 Aix Marseille University, INSERM, Marseille Medical Genetics, Department of Endocrinology, Assistance Publique Hopitaux de Marseille, Marseille, France; 5 Department of Surgery, Huyssens Foundation Clinics, Essen, Germany; 6 Neuroendocrine Tumors Service, Sheba Medical Center and Sackler Faculty of Medicine, Tel Aviv University, Tel Aviv, Israel; 7 Division of General Surgery, Mayo Clinic, Rochester, Minnesota; 8 Department of Oncologic and Urologic Surgery, the 903rd PLA Hospital, Wenzhou Medical University, Hangzhou, Zhejiang, People’s Republic of China; 9 Department of Endocrinology, Seth GS Medical College and KEM Hospital, Mumbai, India; 10 Department of Surgical Oncology, Bakhrushin Brothers Moscow City Hospital, Moscow, Russia; 11 Department of Surgery, Endocrinology Research Center, Moscow, Russia; 12 Department of Endocrinology, University of Groningen, University Medical Center Groningen, Groningen, the Netherlands; 13 Instituto do Cancer do Estado de São Paulo (ICESP), Serviço de Endocrinologia, Hospital das Clínicas (HCFMUSP), Faculdade de Medicina da Universidade de São Paulo, São Paulo, Brazil; 14 Familial Cancer Clinic and Oncoendocrinology, Veneto Institute of Oncology IOV–IRCCS, Padua, Italy; 15 Operative Unit of the Endocrinology Department of Medicine (DIMED), University of Padua, Padua, Italy; 16 Department of Molecular Endocrinology, Institute of Endocrinology, Prague, Czech Republic; 17 Department of Endocrine Oncology and Nuclear Medicine, Maria Sklodowska-Curie Institute–Oncology Center, Gliwice Branch, Gliwice, Poland; 18 Institute of Endocrinology and Metabolism NAMS of Ukraine, Kiev, Ukraine; 19 Institute of Cardiology, Department of Hypertension, Warsaw, Poland; 20 Department of Endocrinology, Ospedale Niguarda Cà Granda, Milan, Italy; 21 Department of Medicine, Division of Endocrinology, Tufts Medical Center, Boston, Massachusetts; 22 Department of Radiology, Medical Center–University of Freiburg, Faculty of Medicine, University of Freiburg, Freiburg, Germany; 23 Department of Urology, Xiangya Hospital, Central South University, Changsha, China; 24 Department of Biochemistry and Molecular Biology, School of Life Sciences, Central South University, Changsha, China; 25 Hospital Universitari de Girona, Gerencia Territorial Girona, Institut Català de la Salut, Girona, Spain; 26 Endocrine Section, Hospital del Salvador, Santiago de Chile, Department of Medicine University of Chile, Santiago, Chile; 27 Department of Endocrinology and Metabolic Diseases, Leiden University Medical Center, Leiden, the Netherlands; 28 Department of Endocrinology, E.E. Eichwald Clinic, I.I. Mechnikov Northwestern State Medical University, St Petersburg, Russia; 29 Department of Molecular Medicine and Surgery, Karolinska Institutet, Stockholm, Sweden; 30 Neuroendocrine Tumor Unit, Endocrinology and Metabolism Service, Department of Medicine, ENETS Centre of Excellence, Hadassah-Hebrew University Medical Center, Jerusalem, Israel; 31 Division of Endocrinology and Metabolism, Siriraj Hospital, Mahidol University, Bangkok, Thailand; 32 Endocrinology, Abdominal Center, University of Helsinki and Helsinki University Hospital, Helsinki, Finland; 33 Department of Nuclear Medicine and Endocrinology, Second Faculty of Medicine, Charles University in Prague and Motol University Hospital, Prague, Czech Republic; 34 Department of Medicine II, Medical Center–University of Freiburg, Faculty of Medicine, University of Freiburg, Freiburg, Germany; 35 Genomic Medicine Institute, Lerner Research Institute and Taussig Cancer Institute, Cleveland Clinic, Cleveland, Ohio

## Abstract

**Importance:**

Large studies investigating long-term outcomes of patients with bilateral pheochromocytomas treated with either total or cortical-sparing adrenalectomies are needed to inform clinical management.

**Objective:**

To determine the association of total vs cortical-sparing adrenalectomy with pheochromocytoma-specific mortality, the burden of primary adrenal insufficiency after bilateral adrenalectomy, and the risk of pheochromocytoma recurrence.

**Design, Setting, and Participants:**

This cohort study used data from a multicenter consortium-based registry for 625 patients treated for bilateral pheochromocytomas between 1950 and 2018. Data were analyzed from September 1, 2018, to June 1, 2019.

**Exposures:**

Total or cortical-sparing adrenalectomy.

**Main Outcomes and Measures:**

Primary adrenal insufficiency, recurrent pheochromocytoma, and mortality.

**Results:**

Of 625 patients (300 [48%] female) with a median (interquartile range [IQR]) age of 30 (22-40) years at diagnosis, 401 (64%) were diagnosed with synchronous bilateral pheochromocytomas and 224 (36%) were diagnosed with metachronous pheochromocytomas (median [IQR] interval to second adrenalectomy, 6 [1-13] years). In 505 of 526 tested patients (96%), germline mutations were detected in the genes *RET* (282 patients [54%]), *VHL* (184 patients [35%]), and other genes (39 patients [7%]). Of 849 adrenalectomies performed in 625 patients, 324 (52%) were planned as cortical sparing and were successful in 248 of 324 patients (76.5%). Primary adrenal insufficiency occurred in all patients treated with total adrenalectomy but only in 23.5% of patients treated with attempted cortical-sparing adrenalectomy. A third of patients with adrenal insufficiency developed complications, such as adrenal crisis or iatrogenic Cushing syndrome. Of 377 patients who became steroid dependent, 67 (18%) developed at least 1 adrenal crisis and 50 (13%) developed iatrogenic Cushing syndrome during median (IQR) follow-up of 8 (3-25) years. Two patients developed recurrent pheochromocytoma in the adrenal bed despite total adrenalectomy. In contrast, 33 patients (13%) treated with successful cortical-sparing adrenalectomy developed another pheochromocytoma within the remnant adrenal after a median (IQR) of 8 (4-13) years, all of which were successfully treated with another surgery. Cortical-sparing surgery was not associated with survival. Overall survival was associated with comorbidities unrelated to pheochromocytoma: of 63 patients who died, only 3 (5%) died of metastatic pheochromocytoma.

**Conclusions and Relevance:**

Patients undergoing cortical-sparing adrenalectomy did not demonstrate decreased survival, despite development of recurrent pheochromocytoma in 13%. Cortical-sparing adrenalectomy should be considered in all patients with hereditary pheochromocytoma.

## Key Points

**Question:**

Is cortical-sparing adrenalectomy associated with increased pheochromocytoma-specific morbidity and mortality for patients with bilateral pheochromocytomas compared with total adrenalectomy?

**Findings:**

In this cohort study of 625 patients with bilateral pheochromocytomas, most had hereditary syndromes, but 36% initially presented with unilateral pheochromocytoma. Bilateral total adrenalectomy resulted in a high rate of adverse effects from glucocorticoid replacement therapy, whereas cortical-sparing surgery was not associated with a worse outcome.

**Meaning:**

These findings suggest that cortical-sparing surgery may be the preferred approach for patients at risk for, or diagnosed with, bilateral pheochromocytomas, especially those harboring a germline mutation in one of the known predisposition genes.

## Teaser

This cohort study compares outcomes associated with cortical-sparing adrenalectomy vs total adrenalectomy for patients with bilateral pheochromocytomas.

## Introduction

Pheochromocytomas are tumors that store and release catecholamines in excess, leading to episodes of hypertension, profuse sweating, headaches, panic attacks, arrhythmia, stroke, and death.^1^ According to the World Health Organization classification, the term *pheochromocytoma* is reserved for adrenal location, whereas similar tumors outside the adrenals are named paragangliomas.^2^ Bilateral pheochromocytomas may present either synchronously or metachronously. Bilateral pheochromocytomas are often heritable and have been shown to occur mainly in patients with multiple endocrine neoplasia type 2 (MEN 2) caused by germline mutations of the *RET* (rearranged during transfection*)* proto-oncogene, von Hippel-Lindau disease (VHL; *VHL* gene) and the paragangliomas syndromes types 1 and 4 caused by mutations in the succinate dehydrogenase (*SDH*) subunit D (*SDHD)* and B (*SDHB)* genes, respectively.^3,4,5^ Other less common genes associated with pheochromocytoma include neurofibromatosis type 1 (*NF1), SDHA, SDHC,* SDH assembly factor 2 (*SDHAF2),* transmembrane protein 127 *(TMEM127),* MYC-associated factor X (*MAX),* and several recently reported genes, such as *SLC25A11*, *FH*, and *MDH2*.^6,7,8,9,10,11,12^

The standard treatment of pheochromocytoma is resection.^13^ However, in bilateral pheochromocytomas, the removal of the tumor only vs the entire adrenal gland remains an open question. The 2014 Endocrine Society management guidelines^14^ recommend cortical-sparing adrenalectomy for bilateral and hereditary pheochromocytoma based on low grade of evidence. Although following guidelines in specialized centers demonstrated favorable short-term outcomes, uncertainty remains because of the greater than 10% risk of metastatic pheochromocytomas and the potential of developing new ipsilateral pheochromocytomas.^15,16^

We sought to compare outcomes of total and cortical-sparing adrenalectomy in a large cohort of patients with bilateral pheochromocytoma with objectives to (1) describe the reasons for recommending total vs cortical-sparing adrenalectomy, (2) determine the burden of steroid dependency, (3) determine the risk of pheochromocytoma recurrence after cortical-sparing adrenalectomy, and (4) determine the outcome of total vs cortical-sparing adrenalectomy on pheochromocytoma-specific mortality.

## Methods

We established the European-American-Asian-Bilateral-Pheochromocytoma-Registry, which included patients who underwent surgery for bilateral pheochromocytomas either simultaneously or by subsequent procedures. The institutional review boards for human subjects’ protection or ethical committees of all participating centers approved this study. Patients provided written informed consent or consent waiver, according to the local protocols. In The Netherlands, data were collected anonymously, and no further ethical approval was required. This study followed the Strengthening the Reporting of Observational Studies in Epidemiology (STROBE) reporting guideline for cohort studies. Data were analyzed from September 1, 2018, to June 1, 2019.

The registry was initiated on May 1, 2018, and was based on our International-MEN-2-Pheochromocytoma-Registry and our prospectively accrued European-American-Asian-Pheochromocytoma-Paraganglioma-Registry.^7,17^ It was open to any additional center and any patients with bilateral pheochromocytoma, which resulted in the number of registrants nearly doubling. The project was performed as an international, multicenter cohort study with retrospective examination of data collected from a prospective clinical protocol on which all centers collaborated. All centers recontacted the patients; thus, 57% of the registrants’ information was updated in 2018 (eTable 1 in the Supplement).

We systematically registered demographic, clinical, and molecular genetic data that included the year of operations on adrenals and the size, location, and number of tumors. We also collected data on surgical treatment. We considered cortical-sparing adrenalectomy unsuccessful when the patient became steroid dependent.

The diagnosis of surgically induced hypocortisolism was based on the cortisol measurements (with or without cosyntropin stimulation test) after surgery. In steroid-dependent patients, we registered the doses of glucocorticoid and mineralocorticoid replacement therapy. We verified whether there were episodes of undertreatment (eg, weakness, skin pigmentation, anorexia, vomiting, nausea, orthostatic hypotension, and/or hypoglycemia). We registered the number of adrenal crises (defined as acute hypotension, fatigue, progressive weakness, and abdominal discomfort necessitating high doses of glucocorticoids), and we searched for events that incited the crises. We registered signs of glucocorticoid overtreatment (iatrogenic Cushing syndrome) as new-onset diabetes, obesity, weakness or fatigability, osteoporosis, and fractures. In all patients, we registered the development of ipsilateral recurrence and whether removal resulted in steroid dependency as well as occurrence of malignancy. If an individual was deceased, the cause of death was retrieved.

All patients were offered genetic counseling and molecular genetic analyses for the pheochromocytoma susceptibility genes, including *RET, VHL, MAX, SDHA, SDHB, SDHC, SDHD, SDHAF2, TMEM127,* and *NF1*. We performed Sanger sequencing for all exons of these genes and searched for large deletions or rearrangements by multiplex ligation-dependent probe amplification in *VHL, MAX, SDHA, SDHB, SDHC, SDHD, SDHAF2, *and *TMEM127.* For neurofibromatosis type 1 (NF-1)*,* presence of skin neuromas was regarded as equivalent to mutation positivity.^18^ Registrants were classified as mutation negative if they had no pathogenic DNA variant in these 9 genes and no clinical evidence for NF-1.^19^ The results of molecular genetic analysis and the protocols of molecular genetic screening were evaluated by one of us (F.S.) (eTable 2 in the Supplement).

### Statistical Analysis

Continuous data are presented as median and interquartile range (IQR), while categorical variables are presented as absolute and relative frequencies. We performed statistical comparisons of quantitative data with student *t* tests or analysis of variances. For statistical comparisons of dichotomous data, we used the χ^2^ test. All statistical tests were 2-sided with *P* < .05 considered statistically significant.

## Results

The European-American-Asian-Bilateral-Pheochromocytoma-Registry, a collaboration of 45 centers from 19 countries, included a total of 625 patients (300 [48%] female) as of December 31, 2018. The median (IQR) age at diagnosis with pheochromocytoma was 30 (22-40) years (Table 1). Initial diagnosis of pheochromocytoma occurred between 1950 and 2018. Of 625 patients, 401 (64%) were diagnosed with synchronous bilateral pheochromocytomas and 224 (36%) with metachronous pheochromocytomas, with the contralateral pheochromocytoma diagnosed after a median (IQR) interval of 6 (1-13) years.

**Table 1.** Demographic and Genetic Data of All Registrants

Characteristic	No. (%)			*P* Value
	Total (N = 625)	Steroid Dependent (n = 377)	Steroid Independent (n = 248)	
General features				
Female	301 (48.2)	195 (51.7)	106 (42.7)	.03
Age at diagnosis, y				<.001
Median (IQR)	30 (22-41)	31 (25-42)	27 (18-40)	
95% CI		31.89-34.55	27.99-31.83	
Gene mutated				
* RET*	282 (53.6)	207 (69.9)	75 (32.6)	<.001
* VHL*	184 (35.0)	74 (25.0)	110 (47.8)	<.001
* NF1*	17 (3.2)	9 (3.0)	8 (3.5)	.60
* MAX*	9 (1.7)	3 (1.0)	6 (2.6)	.09
* SDHD*	7 (1.4)	0	7 (3.0)	.001
* TMEM127*	5 (0.9)	2 (0.7)	3 (1.4)	.40
* SDHB*	1 (0.2)	1 (0.4)	0	.40
No mutation found	21 (4.0)	0	21 (9.1)	<.001
Genetic syndromes by clinical data				
Total	469 (75.2)	316 (83.8)	153 (61.6)	<.001
Other syndromic tumor in proband	348 (55.4)	250 (65.7)	98 (39.5)	<.001
Family history of a syndrome	290 (46.4)	188 (49.8)	102 (41.1)	.06

Abbreviation: IQR, interquartile range.

Information on the size of pheochromocytoma was available based on computed tomography scans and/or magnetic resonance imaging for 919 adrenal glands, which demonstrated single small pheochromocytoma with a mean diameter of up to 2.5 cm in 330 glands (36%), medium-sized pheochromocytoma of 2.6 to 5.0 cm in 364 glands (40%), large pheochromocytoma of greater than 5.0 cm in 157 glands (17%), and multiple pheochromocytomas in 68 glands (7%). In addition to adrenal pheochromocytomas, 66 patients had paragangliomas, which were not included in further analyses.

Of 849 adrenalectomies performed in 625 patients, 324 (52%) were planned as cortical sparing and were successful in 248 of 324 patients (76.5%). Steroid dependency developed in 377 patients, 301 of whom had been treated with total adrenalectomy and 76 of whom had undergone unsuccessful cortical-sparing adrenalectomy (Figure). Synchronous bilateral adrenalectomy was performed in 401 patients (64%), whereas 224 patients (36%) had a second adrenalectomy for a contralateral pheochromocytoma at a median (IQR) of 8 (4-13) years after the first surgery. Of 401 patients undergoing synchronous bilateral adrenalectomies, 193 (48%) were treated with cortical-sparing adrenalectomy. In contrast, of 224 patients who underwent metachronous bilateral adrenalectomy, 131 (58%) had a cortical-sparing operation, although only 62 (28%) had cortical-sparing adrenalectomy at first presentation. Primary adrenal insufficiency occurred in all patients treated with total adrenalectomy but only in 23.5% of patients treated with attempted cortical-sparing adrenalectomy. A third of patients with adrenal insufficiency developed complications, such as adrenal crisis or iatrogenic Cushing syndrome.

**Figure.**
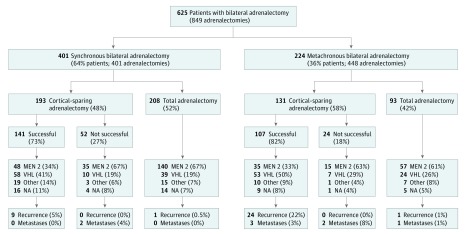
Schema for Bilateral Pheochromocytoma Registrants, Their Operations, and Steroid-Dependent vs Steroid-Independent Outcomes MEN 2 indicates patients with medullary thyroid carcinoma plus pheochromocytoma and/or patients with a *RET* mutation; VHL, pheochromocytoma with hemangioblastoma of eyes or central nervous system and/or patients with a *VHL* mutation; other, patients with a mutation in one of the genes *SDHD*,* SDHB*,* MAX*, or *TMEM127* or signs of neurofibromatosis type 1 or clinically familial pheochromocytomas.

Of 849 adrenalectomies, 395 (47%) were endoscopic operations performed in 322 patients; 44% were performed laparoscopically through frontal approach, 46% through retroperitoneal posterior approach, and 10% through retroperitoneal lateral approach.

Patients treated with cortical-sparing adrenalectomy presented with smaller size of pheochromocytoma (median size, 30 mm; 95% CI, 30.14-34.37 mm) compared with patients treated with total adrenalectomy (median size, 35 mm; 95% CI, 34.54-40.05 mm) (*P* < .001). Success of cortical-sparing adrenalectomy was not associated with size (successful: median size, 30 mm; 95% CI, 29.55-34.27 mm vs not successful: median size, 31 mm; 95% CI, 33.96-38.72 mm; *P* = .60).

Patients were more likely to be treated with cortical-sparing adrenalectomy since 2010 (135 of 225 [60%] vs 189 of 400 [47%]; *P* = .002). Moreover, cortical-sparing adrenalectomy was more likely to be successful in 2010 or later compared with before 2010 (156 of 189 [83%] vs 92 of 135 [68%]; *P* = .003). Certain centers were more likely to proceed with cortical-sparing adrenalectomy; 178 of 324 cortical-sparing adrenalectomies (55%) were performed in Germany, as opposed to only 146 of 324 (45%) in other centers (difference, 10%; 95% CI, 7%-14%; *P* < .001).

### Genetics

Mutation analysis was performed for all susceptibility genes (*RET, VHL, MAX, SDHA, SDHB, SDHC, SDHD, SDHAF2,* and *TMEM127)* in 526 patients (84%) and germline mutations were detected in 505 patients (96%). Mutations of *RET* were present in 282 patients, *VHL* in 184, *NF1* in 17, *MAX* in 9, *SDHD* in 7, *TMEM127* in 5, and *SDHB* in 1 (Table 1). In contrast, clinical evidence of heritability by patient history and family history was positive in only 469 of 625 patients (75%). A total of 576 of 625 patients (92%) carried at least 1 clinical hallmark of heredity, such as any neoplasia that is a component of pheochromocytoma syndromes. These included 48 patients with clinical evidence of MEN 2 (of 330 total patients with MEN 2), 7 patients with VHL-associated hemangioblastomas (of 191 total patients with VHL), and 14 patients with relatives who had pheochromocytomas.

### Outcomes

Follow-up data were available for 558 patients for a median (IQR) of 8 (3-25) years. Of 377 patients who became steroid dependent, 301 (80%) underwent complete bilateral adrenalectomy and 76 (20%) had an attempted but unsuccessful cortical-sparing surgery (Figure and Table 2). Of the 248 steroid-independent patients, all had successful cortical-sparing operations (Figure). Patients with MEN 2 were more likely to present with synchronous bilateral pheochromocytoma compared with patients with VHL (222 of 330 [67%] vs 108 of 191 [57%]; difference, 10%; 95% CI, 2%-19%; *P* = .01) and were more frequently treated with total adrenalectomy (198 of 330 [60%] vs 62 of 191 [32%]; difference, 28%; 95% CI, 19%-36%; *P* < .001). When cortical-sparing adrenalectomy was attempted, it was less likely to be successful in patients with MEN 2 compared with patients with VHL (82 of 132 [62%] vs 112 of 129 [87%]; difference, 25%; 95% CI, 14%-34%; *P* < .001). Metastatic pheochromocytoma occurred in 4 of 191 patients with VHL (2%), as opposed to 1 of 330 patients with MEN 2 (0.3%) (difference, 2%; 95% CI, 0%-5%; *P* = .06). Patients with VHL were at higher risk for development of recurrent ipsilateral pheochromocytoma compared with patients with MEN 2 overall (23 of 191 [12%] vs 8 of 330 [2%]; difference, 10%; 95% CI, 5%-15%; *P* < .001), as well as among all patients undergoing cortical-sparing adrenalectomy only (21 of 129 [16%] vs 8 of 132 [6%]; difference, 10%; 95% CI, 3%-18%; *P* = .01).

**Table 2.** Outcome Data for All Registrants

Outcome	No./Total No. (%)			*P* Value
	Total (n = 625)	Steroid Dependent (n = 377)	Steroid Independent (n = 248)	
Follow-up, median (IQR), y	8 (3-25)	10 (4-22)	7 (3-13)	<.001
Relapse in ipsilateral adrenal	35/625 (5.6)	2/377 (0.5)	33/248 (13.3)	<.001
Adrenal crises at least once	67/625 (10.7)	67/377 (17.7)	NA	NA
No. of adrenal crises	177/625 (28.3)	177/377 (46.9)	NA	NA
No. of patients with metastases of any primary	47/625 (7.5)	37/377 (9.8)	10/248 (4.0)	.001
No. of deaths	63/625 (10.1)	47/377 (12.4)	16/248 (6.4)	<.001
Symptoms of steroid overdosing	50/625 (8.0)	50/377 (13.2)	NA	NA
Causes of adrenal crises				
Infection	45/67 (67.2)	45/67 (67.2)	NA	NA
Stress	14/67 (20.9)	14/67 (20.9)	NA	NA
Trauma	2/67 (3.0)	2/67 (3.0)	NA	NA
Withdrawal or noncompliance	6/67 (9.0)	6/67 (9.0)	NA	NA
Metastatic tumors in living patients				
Pheochromocytoma or paraganglioma	8/37 (21.6)	5/29 (17.2)	3/8 (37.5)	.90
Multiple endocrine neoplasia type 2 with medullary thyroid carcinoma	18/37 (48.7)	18/29 (62.1)	0/8	.001
VHL with renal cell carcinoma	2/37 (5.4)	2/29 (6.9)	0/8	.50
VHL with pancreatic neuroendocrine tumor	7/37 (18.9)	3/29 (10.3)	4/8 (50)	.40
VHL with other	0/37	0/29	0/8	>.99
Nonsyndromic cancer	2/37 (5.4)	1/29 (3.5)	1/8 (12.5)	.80
Causes of deaths				
Pheochromocytoma or paraganglioma	3/63 (4.8)	2/47 (4.3)	1/16 (6.25)	>.99
Adrenal crises	2/63 (3.2)	2/47 (4.3)	NA	NA
Multiple endocrine neoplasia type 2 with medullary thyroid carcinoma	31/63 (49.2)	25/47 (53.2)	6/16 (37.5)	.001
VHL with renal cell carcinoma	1/63 (1.6)	1/47 (2.1)	0/16	>.99
VHL with pancreatic neuroendocrine tumor	3/63 (4.8)	1/47 (2.1)	2/16 (12.5)	.50
VHL with other	3/63 (4.8)	2/47 (4.3)	1/16 (6.3)	>.99
Nonsyndromic cancer	8/63 (12.7)	6/47 (12.8)	2/16 (12.5)	>.99
Other cause or unknown	12/63 (19.1)	8/47 (17.0)	4/16 (25.0)	>.99

Abbreviations: IQR, interquartile range; NA, not applicable; VHL, von Hippel-Lindau disease.

Recurrent ipsilateral pheochromocytoma developed in 35 of 625 patients (5.6%). Ipsilateral recurrence after successful cortical-sparing adrenalectomy occurred in 33 of 248 patients (13%) after a median (IQR) of 8 (4-17) years, as well as 2 of 301 patients after medians of 9 and 25 years after total adrenalectomy (difference, 13%; 95% CI, 9%-17%; *P* < .001) (Figure). Of the 33 patients who developed ipsilateral recurrence after successful cortical-sparing adrenalectomy, 10 (30%) became steroid dependent after a second surgery. None of the patients treated with unsuccessful cortical-sparing adrenalectomy experienced recurrence (0 of 76 [0%] vs 33 of 248 [13%]; difference, 13%; 95% CI, 7%-18%; *P* < .001).

Metastatic pheochromocytoma developed in 8 of 625 patients (1.3%), including 1 patient treated with total adrenalectomy and 7 patients treated with cortical-sparing adrenalectomy (1 of 301 [0.3%] vs 7 of 324 [2%]; difference, 2%; 95% CI, 0%-4%; *P* = .07). Metastatic pheochromocytoma was more common in patients with metachronous pheochromocytomas (6 of 224 [2.7%] vs 2 of 401 [0.5%]; difference, 2%; 95% CI, 0.2%-5%; *P* = .03). One of the 8 patients with metastatic pheochromocytoma was diagnosed prior to adrenalectomy, and in another 3 patients, the origin of metastases was likely a coexisting paraganglioma. Of the 8 patients with metastatic pheochromocytoma, 4 patients had germline mutation in *VHL*, 1 in *MAX*, and 1 in *RET*.

An additional 269 patients developed malignant neoplasms other than pheochromocytoma. These included medullary thyroid carcinoma (MTC) in 223 of 330 (68%) with MEN 2. Renal cell carcinoma and pancreatic neuroendocrine tumors were seen in 37 of 191 patients (19%) with VHL. Nine further patients had malignant neoplasms not associated with MEN 2, VHL, or pheochromocytoma syndromes.

### Complications of Steroid Replacement

Patients became steroid dependent at a median (IQR) age of 34 (27-43) years; 41 of 377 patients (11%) were younger than 20 years. They were treated with a median (IQR) daily dose of hydrocortisone of 30 (20-30) mg; in addition, 294 patients (78%) also required fludrocortisone at a median (IQR) daily dose of 0.05 (0.05-0.1) mg. Median (IQR) duration of steroid use was 13 (4-20) years.

Of 377 steroid-dependent patients, 67 (18%) developed at least 1 adrenal crisis; 22 patients (6%) had 2 or more adrenal crises (total, 177 events). Reasons for adrenal crisis development were infections (68%), stress (20%), noncompliance or medication errors (9%), and trauma (3%).

During follow-up, 50 patients (13%) developed symptoms consistent with steroid overreplacement. Of these 50, 21 (42%) were treated with hydrocortisone doses of more than 25 mg daily, whereas 29 patients (58%) were treated with physiological hydrocortisone replacement.

### Overall Survival

Overall survival was associated with comorbidities unrelated to pheochromocytoma: among 63 patients who died, only 3 (5%) died of metastatic pheochromocytoma.

Cortical-sparing surgery was not associated with pheochromocytoma-specific survival. During median (IQR) follow-up of 8 (4-17) years, overall survival of steroid-dependent patients was significantly lower compared with steroid-independent patients (85% vs 99%; difference, 14%; 95% CI, 11%-18%; *P* = .01); however, most of the difference was due to higher rates of MEN 2 (and metastatic MTC) in steroid-dependent patients. Of the 47 steroid-dependent patients who died, 25 (53%) died of metastatic MTC, 2 (4%) of metastatic pheochromocytoma, 2 (4%) of adrenal crises, 4 (8%) of VHL complications, and 14 (30%) of morbidity unrelated to pheochromocytoma syndromes. In contrast, of the 16 steroid-independent patients who died, 1 (6%) had metastatic pheochromocytoma, 6 (38%) had metastatic MTC, 3 (19%) had VHL-related complications, and 6 (38%) died of unrelated causes.

## Discussion

Patients with bilateral pheochromocytomas are frequently treated with total bilateral adrenalectomy. While cortical-sparing adrenalectomy was introduced in practice in 1999, it is still a relatively underused procedure.^20,21,22,23^ A recent meta-analysis^16^ reported that cortical-sparing adrenalectomy can reduce the need for steroid replacement therapy and carries a low risk for recurrence, based on mainly retrospective studies of small sample size. Overall, the evidence for use of cortical-sparing adrenalectomy in patients with bilateral pheochromocytomas is minimal, leading to only a weak recommendation in the recent guidelines on management of pheochromocytoma.^14,16^ In this article, we present data on outcomes of treatment in patients with bilateral pheochromocytomas based on a retrospective-prospective series of 625 patients enrolled in 19 countries followed up for a median of 8 years. A large number of patients undergoing cortical-sparing adrenalectomy allowed us to perform subgroup analyses for both common and rare outcomes, such as risk of metastatic or recurrent disease, risk of adrenal insufficiency, and complications of steroid replacement therapy.

In our cohort of patients treated with cortical-sparing adrenalectomy, recurrent pheochromocytoma in the spared ipsilateral adrenal gland occurred in 13% of patients, mostly in patients with VHL and MEN 2, similar to the reported 8% to 10% in other studies.^16,24^ Recurrence occurred as little as 1 year and as much as 27 years after cortical-sparing adrenalectomy. As several patients were diagnosed with early recurrence, it is likely that this was a case of missed second pheochromocytoma rather than a true recurrence. In all these patients, recurrent tumors were successfully removed, and most patients remained steroid independent, thus avoiding or delaying the onset of adrenal insufficiency.

As expected, all patients undergoing bilateral total adrenalectomy became steroid dependent. However, we also found that 23% of patients with cortical-sparing adrenalectomy developed adrenal insufficiency, requiring lifelong steroid replacement (Figure). The reasons for adrenal insufficiency developing in patients with spared adrenals could be insufficient blood supply to the remaining adrenal gland or inadequate remaining cortical tissue. In steroid-dependent patients, complications of either disease or treatment were frequent, as 18% of patients developed at least 1 adrenal crisis during follow-up, and manifestations of iatrogenic Cushing syndrome (overreplacement with glucocorticoids) occurred in 13%. As patients were frequently treated with hydrocortisone doses greater than 20 to 25 mg per day, it is possible that the high prevalence of iatrogenic Cushing syndrome in our cohort was also due to a higher daily steroid replacement standard in the past.

It is important to note that the patients undergoing cortical-sparing adrenalectomy had slightly smaller pheochromocytomas in our study. Indeed, total adrenalectomy has been suggested for large pheochromocytomas.^1,25^

As expected, most patients with bilateral pheochromocytoma had a predisposing genetic mutation, as demonstrated in 96% of patients in our cohort with available testing. Molecular genetics can dramatically improve preoperative characterization of patients as having heritable vs sporadic pheochromocytoma.^26^ The most frequent mutations in our cohort involve *RET* leading to MEN 2 and *VHL* leading to VHL disease. Although personal and family histories are important in diagnosing heritable pheochromocytoma, patients with an underlying germline mutation often present without hallmark clinical features suggestive of a particular hereditary syndrome.^4,7,9,27^ Availability of DNA sequencing provides a powerful diagnostic tool to identify such mutations in patients with apparently sporadic pheochromocytoma. This is poignantly illustrated by the fact that 36% of our cohort initially presenting with unilateral pheochromocytoma subsequently developed contralateral disease. In these patients, molecular genetic results could have changed initial management,^17,28,29^ leading to the decision to proceed with a cortical-sparing surgery in patients at high likelihood of future contralateral disease. Moreover, an individualized monitoring approach to include workup for nonpheochromocytoma manifestations of a specific syndrome could have been instituted at an earlier time.

We found that steroid-dependent patients demonstrated a higher mortality when compared with patients with preserved adrenal function; notably, this increased mortality was mainly attributed to metastatic MTC in patients with MEN 2, who were overrepresented among patients with primary adrenal insufficiency. However, it is important to note that in 2 patients, adrenal crisis was the main cause of death.

Patients with pheochromocytoma, and especially hereditary pheochromocytoma, benefit from an expert multidisciplinary team including an adrenal endocrinologist, surgeon, oncologist, and geneticist. Adrenal surgical expertise is not widely available, with less than one-third of all surgeons performing more than 4 adrenalectomies per year.^30^ Cortical-sparing adrenalectomy necessitates even higher surgical expertise and should be performed by a high-volume adrenal surgeon.

Cortical-sparing adrenalectomy may be performed during open or minimally invasive adrenalectomy. While there is wide variation regarding the technical aspects of this complex procedure, certain suggestions can be reasonably made. Prior to mobilization and devascularization of the pheochromocytoma, intraoperative ultrasonography should be performed to define the anatomy of the pheochromocytoma relative to the remaining normal adrenal gland and the adrenal vein. This will optimize operative planning and allow the surgeon to devascularize only the portion of adrenal that is to be resected. Furthermore, ultrasonography will help exclude additional ipsilateral pheochromocytomas, which may be small and unexpected based on preoperative imaging. Extracapsular dissection and mobilization of the pheochromocytoma should be performed, ensuring not to rupture the capsule, which can result in recurrence. A remnant one-third the size of a normal adrenal gland is adequate for appropriate synthetic function.^23,31,32^

### Limitations

This study had several limitations. The retrospective portion of the study did not allow for a uniform data collection for certain variables; however, this allowed for longitudinal follow-up and a large sample size otherwise impossible in a purely prospective study of this rare disease. Variability in local protocols and/or local surgical expertise likely influenced the choice of therapy. The surgical approach was decided by the surgeon based on multiple factors, including tumor size and the ability to secure vascular supply to the remaining adrenal cortex. Given the extensive period for patient enrollment, certain approaches changed over the years, including choice of surgical procedures as well as glucocorticoid replacement therapy.

## Conclusions

This study found that undergoing cortical-sparing adrenalectomy for bilateral pheochromocytomas was not associated with decreased survival, suggesting that it should be considered in patients with hereditary pheochromocytoma. Based on our findings, we suggest consideration of several factors when selecting an appropriate surgical procedure for a patient with pheochromocytoma. Cortical-sparing adrenalectomy should be considered in patients with (1) bilateral pheochromocytomas, (2) tumor size less than 5 cm, or (3) high likelihood of a predisposing mutation leading to high risk of metachronous pheochromocytoma in the future. Our study suggests that cortical-sparing surgery for pheochromocytoma is appropriate and safe in the right setting. All patients with pheochromocytomas, even when presenting with apparently sporadic, unilateral disease, should be offered genetic analysis preoperatively to ensure gene-informed surgical and medical management. Referral to highly specialized adrenal centers for multidisciplinary assessment and treatment should be considered, especially in patients with hereditary pheochromocytoma syndromes.

## Notes

Supplement. **eTable 1.** Provenience of 625 Patients (377 Steroid Dependent and 248 Steroid Independent) **eTable 2.** Germline Mutations in the Genes *RET, VHL, TMEM127, MAX, SDHD, SDHB* and *NF1*
