# Supplementary material for: Comparison of Pheochromocytoma-Specific Morbidity and Mortality Among Adults With Bilateral Pheochromocytomas Undergoing Total Adrenalectomy vs Cortical-Sparing Adrenalectomy
Source: JAMA Netw Open. 2019 Aug 9;2(8):e198898. doi: 10.1001/jamanetworkopen.2019.8898 (PMC6692838; doi:10.1001/jamanetworkopen.2019.8898)
Supplement: Supplement. — eTable 1. Provenience of 625 Patients (377 Steroid Dependent and 248 Steroid Independent) eTable 2. Germline Mutations in the Genes RET, VHL, TMEM127, MAX, SDHD, SDHB and NF1 [file jamanetwopen-2-e198898-s001.pdf]

Supplementary Online Content

Neumann HPH, Tsoy U, Bancos I, et al; International Bilateral-Pheochromocytoma-Registry Group. Comparison of pheochromocytoma-specific morbidity and mortality among adults with bilateral pheochromocytomas undergoing total adrenalectomy vs cortical-sparing adrenalectomy. *JAMA Netw Open*. 2019;2(8):e198898.
doi:10.1001/jamanetworkopen.2019.8898

eTable 1. Provenience of 625 Patients (377 Steroid Dependent and 248 Steroid Independent)

eTable 2. Germline Mutations in the Genes *RET, VHL, TMEM127, MAX, SDHD, SDHB* and *NF1*

This supplementary material has been provided by the authors to give readers additional information about their work.

© 2019 Neumann HPH et al. *JAMA Network Open.*

eTable 1 - Provenience of 625 patients (377 steroid dependent and 248 steroid independent). As pathogenic were called DNA variants as ACMG class 3 or less.

Country	All	Steroid dependent	Steroid Independent
Germany, n (%)	188 (30.1)	20 (5.3)	163 (65.9)
USA, n (%)	96 (15.4)	84 (22.3)	12 (4.9)
China, n (%)	44 (7.1)	14 (3.8)	30 (12.3)
Brazil, n (%)	41 (6.5)	40 (10.8)	1 (0.4)
Italy, n (%)	39 (6.3)	21 (5.6)	18 (7.3)
Netherlands, n (%)	30 (4.8)	30 (7.9)	0 (0)
Russia, n (%)	34 (5.5)	31 (8.3)	3 (1.3)
Poland, n (%)	29 (4.5)	24 (6.5)	5 (2.1)
India, n (%)	27 (4.3)	19 (5.1)	8 (3.4)
France, n (%)	18 (2.9)	16 (4.3)	2 (0.8)
Czech republic, n (%)	17 (2.7)	16 (4.3)	1 (0.4)
Ukraine, n (%)	13 (2.1)	12 (3.2)	1 (0.4)
Spain, n (%)	10 (1.6)	10 (2.7)	0 (0)
Israel, n (%)	9 (1.5)	9 (2.5)	0 (0)
Chile, n (%)	7 (1.1)	7 (1.9)	0 (0)
Sweden, n (%)	7 (1.1)	6 (1.6)	1 (0.4)
Thailand, n (%)	5 (0.8)	5 (1.3)	0 (0)
Turkey, n (%)	5 (0.8)	5 (1.3)	0 (0)
Slovenia, n (%)	3 (0.4)	2 (0.5)	1 (0.4)
Finland, n(%)	2 (0.3)	2 (0.5)	0 (0)
Singapore, n (%)	1 (0.2)	1 (0.3)	0 (0)

© 2019 Neumann HPH et al. *JAMA Network Open.*

eTable 2 Germline mutations in the genes *RET*, *VHL*, *TMEM127*, *MAX*, *SDHD*, *SDHB* and *NF1*

Amino acid change	Nucleotide change	ACMG Variant Class*	Number of Variant Carriers
**RET**			
p.Cys609Phe	c.1826G>T	5	1
p. Cys611Trp	c.1833C>G	4	2
p.Cys618Arg	c.1852T>C	5	3
p.Cys618Ser	c.1852T>A	5	3
p.Cys618Tyr	c.1853G>A	5	2
p.Cys620Arg	c.1858T>C	5	1
p.Cys620Gly	c.1858T>G	5	1
p.Asp631Tyr	c.1891G>T	5	1
p.Cys634Arg	c.1900T>C	5	89
p.Cys634Gly	c.1900T>G	5	27
p.Cys634Phe	c.1901G>T	5	6
p.Cys634Ser	c.1901G>C/c.1900T>A	5	12
p.Cys634Trp	c.1902C>G	5	11
p.Cys634Tyr	c.1901G>A	5	75
p.Val804Leu	c.2410G>C	5	1
p.Val804Met	c.2410G>A	5	1
p.Met918Thr	c.2753T>C	5	46
**VHL**			
p.Leu63Arg	c.188T>G	4	1
p.Arg64Pro	c.191G>C	4	1
p.Ser65Pro	c.193T>C	4	1
p.Ser65Thr	c.193T>A	4	1
p.Ser68Trp	c.203C>G	4	1
p.Ser80Gly	c.238A>G	4	1

© 2019 Neumann HPH et al. *JAMA Network Open.*

p.Pro86Ala	c.256C>G	5	1
p.Pro86Thr	c.256C>A	4	1
p.Pro86Leu	c.257C>T	5	1
p.Gly93Arg	c.277G>C	4	5
p.Gly93Cys	c.277G>T	4	4
p.Gly93Ser	c.277G>A	4	3
p.Tyr98His	c.292T>C	5	25
p.Tyr98Cys	c.293A>G	4	1
p.Tyr98Ser	c.293A>C	4	2
p.Arg107Gly	c.319C>G	4	1
p.Arg107His	c.320G>A	4	2
p.Gly114Ser	c.340G>A	5	1
p.Leu116Pro	c.347T>C	4	1
p.Leu118Arg	c.353T>G	4	1
p.Phe119Leu	c.355T>C	5	2
p.Asp121Gly	c.362A>G	4	1
p.Thr124Ile	c.371C>T	4	4
p.His125Pro	c.374A>C	4	1
p.Thr157Ile	c.470C>T	4	1
p.Leu135*	c.404T>A	5	3
p.Phe136Cys	c.407T>G	4	1
p.Ile151Met	c.453C>G	4	1
p.Tyr156Cys	c.467A>G	4	6
p.Arg161*	c.481C>T	5	1
p.Arg161Gln	c.482G>A	5	21
p.Arg161Pro	c.482G>C	4	2
p.Gln164Arg	c.491A>G	4	3
p.Val166Ala	c.497T>C	4	1
p.Arg167Trp	c.499C>T	5	31

© 2019 Neumann HPH et al. *JAMA Network Open.*

p.Arg167Gln	c.500G>A	5	36
p.Val170Gly	c.509T>G	4	2
p.Leu178Gln	c.533T>A	4	1
p.Leu188Val	c.562C>G	5	1
p.Pro192Leu	c.575C>T	4	2
p.Gln195*	c.583C>T	5	2
p.Leu198Pro	c.593T>C	4	3
p.Leu198Gln	c.593T>A	4	1
p.*214Glyext14	c.640T>G	4	2
**TMEM127**			
p.Ser40Phe	c.119C>T	4	1
p.?	c.410-2A>G	5	1
p.Ser167Profs*10	c.498_499ins350	4	1
p.Phe173Ser	c.518T>C	4	1
p.Gly185Arg	c.553G>A	4	1
**MAX**			
p.Ser52*	c.155C>A	4	1
p.Gln54Lysfs*11	c.160delC	4	1
p.?	c.171+G>A	5	1
p.Arg66*	c.196C>T	5	1
p.Arg75*	c.223C>T	5	2
p.His81Profs*5	c.242_243delAC	4	1
p.Gln98Profs*48	c.292dupC	4	1
p.Glu103*	c.307G>T	4	1
**NF1**			
p.Thr165Leufs*13		4	1

© 2019 Neumann HPH et al. *JAMA Network Open.*

p.Tyr489Cys	c.1466A>G	5	1
p.Gly822*	c.2646G>T	5	3
p.Leu1153Metfs*4	c.3457_3460del	5	1
p.Arg1276*	c.3826C>T	5	1
**SDHD**			
p.Trp5*	c.14G>A	5	3
p.Ala13Profs*55	c.36_37delTG	5	1
p.?	c.52+1G>T	5	1
p.Gln121*	c.361C>T	5	1
Large Deletion		5	1
**SDHB**			
Large Deletion		5	1

*ACMG class 4 (likely pathogenic) or 5 (pathogenic) are considered as mutation positive for purposes of this analysis

© 2019 Neumann HPH et al. *JAMA Network Open.*
